# Structural Biology of LRRK2 and its Interaction with Microtubules

**DOI:** 10.1002/mds.28755

**Published:** 2021-08-23

**Authors:** Andres E. Leschziner, Samara L. Reck‐Peterson

**Affiliations:** ^1^ Department of Cellular and Molecular Medicine University of California San Diego La Jolla California USA; ^2^ Division of Biological Sciences Molecular Biology Section, University of California San Diego La Jolla California USA; ^3^ Division of Biological Sciences, Cell and Developmental Biology Section University of California San Diego La Jolla California USA; ^4^ Howard Hughes Medical Institute Chevy Chase Maryland USA

**Keywords:** LRRK2, structural biology, microtubules, Parkinson's disease, cryo‐EM

## Abstract

Mutations in leucine rich repeat kinase 2 (LRRK2) are a major cause of familial Parkinson's disease (PD) and a risk factor for its sporadic form. LRRK2 hyperactivity has also been reported in sporadic PD, making LRRK2 an appealing target for PD small‐molecule therapeutics. At a cellular level, increasing evidence suggests that LRRK2 regulates membrane trafficking. Under some conditions LRRK2 also associates with microtubules, the cellular tracks used by dynein and kinesin motors to move membranes. At a structural level, however, relatively little was known about LRRK2. An important step toward bridging this gap took place last year with the publication of structures of LRRK2's cytosolic and microtubule‐bound forms. Here, we review the main findings from these studies and discuss what we see as the major challenges going forward with a focus on areas that will require structural information. We also introduce the structural techniques—cryo‐electron microscopy and cryo‐electron tomography—that were instrumental to solving the structures of LRRK2. © 2021 The Authors. *Movement Disorders* published by Wiley Periodicals LLC on behalf of International Parkinson and Movement Disorder Society

## LRRK2: A Brief History and Introduction

*PARK8*, the gene coding for leucine rich repeat kinase 2 (LRRK2), was first linked to familial Parkinson's disease (PD) in the early 2000s.^1^, ^2^ Mutations in *LRRK2* were soon shown to be responsible for the *LRRK2*‐linked familial PD cases,^3^, ^4^ and to be associated with increased risk for the sporadic form of the disease.^5^, ^6^ It was the discovery that these pathogenic PD‐linked mutations lead to the activation of LRRK2's kinase^7^, ^8^, ^9^, ^10^ that turned this protein into a target for small molecule therapeutics to treat familial PD. Interest in LRRK2 as a therapeutic target grew further in 2018, after increased kinase activity in an otherwise wild‐type LRRK2 was seen in postmortem brain tissue from idiopathic PD patients^11^; this made inhibition of LRRK2's kinase activity a potential route to treat all forms of PD. Last year, the first LRRK2‐specific kinase inhibitors successfully completed phase 1b clinical trials (clinicaltrials.gov). Despite this growing interest in LRRK2 as both a target to treat PD and as a window into the cell biology of the disease, until recently, relatively little was known about LRRK2 structure or function. This is in part because of LRRK2's complexity at the molecular and cellular levels.

**FIG 1:**
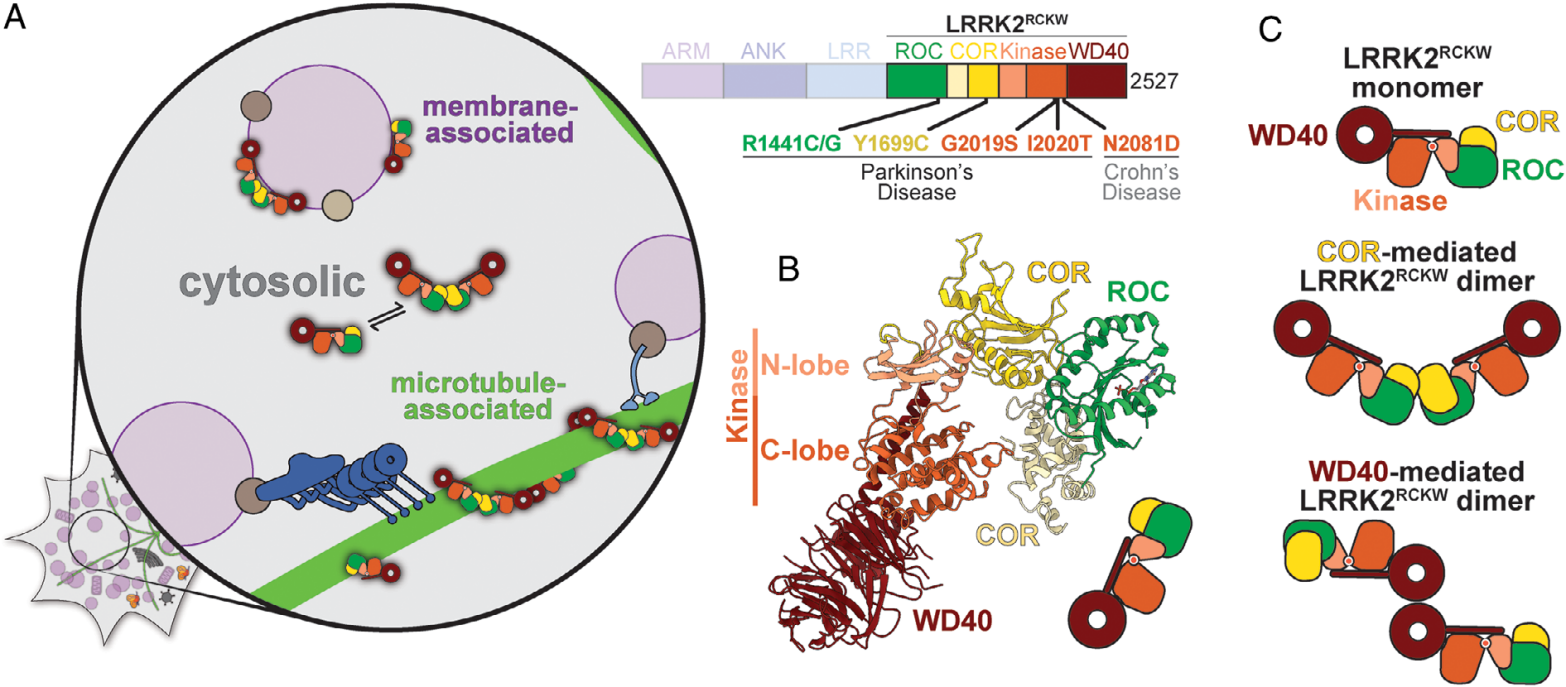
Cryo‐EM structure of LRRK2^RCKW^. (**A**) LRRK2 domain structure (right). The carboxy‐terminal half of LRRK2, referred to as LRRK2^RCKW^ here, is highlighted and contains all of the most common PD mutations and a mutation that is linked to Crohn's disease. The majority of LRRK2 is cytosolic (left). Under some circumstances, LRRK2 associates with membranes (purple) and microtubules (green). LRRK2 is shown in cartoon form based on the structure of LRRK2^RCKW^, with the ROC domain in green, the COR domain in yellow, the kinase domain in orange, and the WD40 domain in red. (**B**) 3.5 Å cryo‐EM structure of LRRK2^RCKW^. LRRK2's kinase is in an open, inactive conformation. (**C**) Cartoon representations of the different LRRK2^RCKW^ structures that have been observed in solution: LRRK2 monomers, COR‐mediated dimers, and WD40‐mediated dimers.

LRRK2 is a 280 kDa multi‐domain protein (Fig. 1A). Its amino‐terminal half is comprised of repetitive protein–protein interaction domains: armadillo, ankyrin, and leucine‐rich repeats, the last one giving rise to the protein's name. LRRK2's carboxy‐terminal half contains two enzymatic activities: a Ras‐like GTPase (Ras of complex, or ROC) and a kinase. This combination of a GTPase and a kinase in the same protein is a unique feature of a subset of members of the Roco family of proteins to which LRRK2 belongs.^12^ The carboxy‐terminal half also contains a structural domain (carboxy‐terminal of ROC, or COR) that separates the GTPase from the kinase, and, at its end, another protein–protein interaction domain, a WD40. All of the most common PD‐linked mutations are found in the carboxy‐terminal half of LRRK2, as well as a mutation that has been linked to Crohn's disease,^13^ which also increases LRRK2's kinase activity.

The bulk of LRRK2 is found in the cytosol of cells.^14^ However, under certain conditions, LRRK2 is also present on intracellular membranes^14^ and microtubules,^8^, ^15^ filaments that serve as the tracks for transporting intracellular cargos (Fig. 1A). Although the physiological relevance of a direct interaction between LRRK2 and microtubules remains to be established, it is clear that LRRK2 plays a role in regulating transport along them. Phosphoproteomics have revealed that 10 different Rab GTPases, which play many roles in membrane biology including in trafficking membranes from one part of the cell to another,^16^ are physiological substrates of LRRK2.^9^, ^17^ Notably, the motors that move along microtubules, dynein and kinesin, bind directly or indirectly to some Rabs.^18^ Finally, four of the five most common PD‐linked mutations in LRRK2 increase its interaction with microtubules when the protein is overexpressed in cells.^15^

LRRK2's complexity also extends to its molecular behavior. LRRK2 has been reported as both a monomer and dimer in solution^8^, ^19^ and as filamentous structures when bound to microtubules.^15^, ^20^ Understanding the cellular function(s) of LRRK2 will require perturbing it at each of its specific locations, probing the consequences of those perturbations, and ultimately determining how this is related to PD. High‐resolution structures of LRRK2 will provide the information needed to do this and to guide the design of LRRK2‐targeted therapeutics.

Until very recently, the only two high‐resolution structures of LRRK2 were of the isolated ROC^21^ and WD40^22^ domains. A number of other structures have also been solved from distant homologs of LRRK2. These include structures of the LRR, ROC, and COR domains from bacterial Roco proteins^23^, ^24^, ^25^ and the kinase domain from the slime mold *Dictyostelium discoideum*'s Roco4.^26^ Although structures of full‐length LRRK2 were previously solved using negative stain electron microscopy^27^ and cryo‐electron microscopy (cryo‐EM),^28^ their limited resolutions prevented a description of the protein at the chemical level.

A major step forward in our understanding of LRRK2 took place last year with the publication of two papers on the structures of its soluble^29^ and microtubule‐associated^20^ forms. The goal of this Perspective is to summarize the main findings from these two studies and their implications for PD and PD therapeutics. Because these structures were solved using cryo‐EM and cryo‐electron tomography (cryo‐ET), which some readers may be less familiar with, we will begin by briefly describing those techniques.

## Cryo‐EM and Cryo‐ET

Although the use of electron microscopy as a tool to determine the three‐dimensional (3D) structure of macromolecules was first demonstrated in 1968,^30^ X‐ray crystallography was the dominant technique for high‐resolution structure determination until recently. Several advances in hardware and software propelled cryo‐EM to the forefront during the past decade, and many excellent reviews have been written on the history of cryo‐EM and what has made its meteoric rise possible (eg, Cheng et al^31^ and Cheng^32^). Currently, cryo‐EM is used mainly to solve high‐resolution structures of purified macromolecules, whereas cryo‐ET is used to obtain 3D reconstructions of far more complex molecular environments, such as the interior of cells. Here, we will briefly introduce cryo‐EM and cryo‐ET and some salient features relevant to the LRRK2 work discussed in this Perspective.

## Cryo‐EM

**FIG 2:**
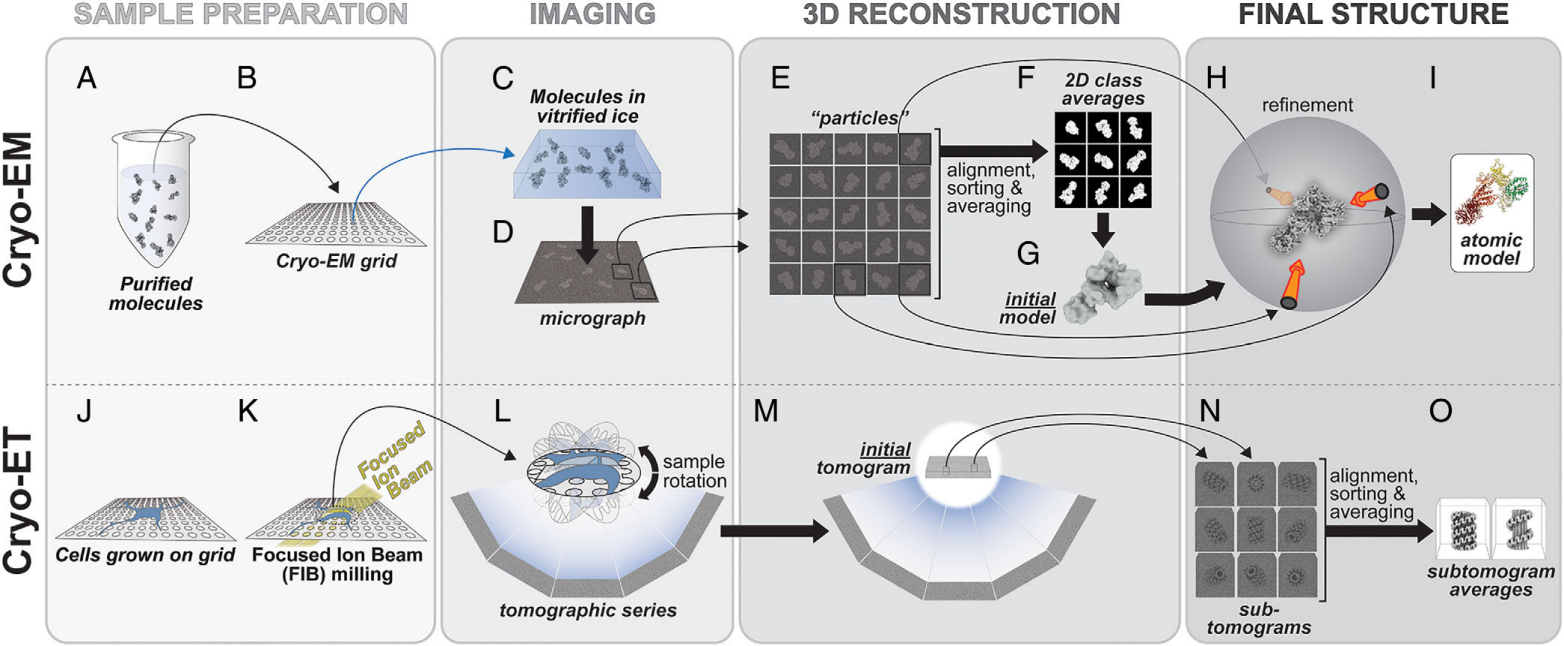
The Cryo‐EM and cryo‐ET pipelines. (**A–I**) In cryo‐EM, Purified molecules (**A**) are placed on EM grids (**B**), frozen (**C**) and imaged using transmission EM (**D**). Individual particles (**E**) are used to generate 2D class averages (**F**), which are then used to build an initial 3D model (**G**) that is then further refined (**H**). Depending on the resolution of the final cryo‐EM map, an atomic model can be built into it (**I**). (**J–O**) In cryo‐ET, cells are grown on EM grids (**J**) and, when targeting thicker areas, FIB milling is used to generate thin slices of cells (**K**). The milled cells are then imaged using transmission EM and a tomographic series is obtained (**L,M**). Sections of a tomogram containing the same structure are segmented to generate sub‐tomograms (**N**) and these are averaged to generate sub‐tomogram averages (**O**). See text for a more detailed explanation of the pipelines.

In cryo‐EM, a solution containing a molecule of interest (Fig. 2A) is applied to a small grid (Fig. 2B), excess liquid is blotted away, leaving a thin layer behind, and the grid is quickly frozen at liquid nitrogen temperatures (Fig. 2C). This rapid freezing is key, because its speed prevents the water in the solution from forming ice crystals, preserving it instead in a vitreous (“glass‐like”) state. The grid is imaged in a transmission electron microscope (Fig. 2D), also at liquid nitrogen temperatures. Because the molecules adopt random orientations within the frozen layer of liquid (Fig. 2C), the images obtained represent many different two‐dimensional (2D) views, or projections, of the molecule, which is precisely what is needed to obtain a 3D reconstruction of its structure. Determining how those views are related spatially is at the heart of cryo‐EM.

A major challenge in cryo‐EM is the sensitivity of biological molecules to electrons; to limit radiation damage, one must minimize the electron dose, resulting in very noisy data (Fig. 2D). This noise must be overcome by collecting (and averaging) many images; a data set may consist of well over a 1000 micrographs, each in turn containing dozens to hundreds of molecules in it. (Individual molecular images are referred to as “particles” and the entire process of reconstructing a molecule's structure as “single particle analysis.”) Once all the individual molecular images have been extracted from the micrographs (Fig. 2E) there are different computational approaches to combine them into a final 3D reconstruction of the molecule. These typically involve three steps. First, the particles are sorted out into groups (“classes”) representing the same view of the molecule, and “class averages” are calculated to improve the signal‐to‐noise ratio (Fig. 2F). Second, the relative orientations of these class averages are calculated computationally to obtain the first (low resolution) representation of the molecule (the “initial model”) (Fig. 2G). Finally, this initial model is used as a reference to determine the spatial orientation of each particle in the data set (Fig. 2H), after which a new structure is calculated. This process, known as “refinement,” is iterated until the resolution of the structure no longer improves. Once a high‐resolution map has been obtained, a molecular model can be built into it (Fig. 2I).

A unique feature of cryo‐EM is its ability to determine the structures of multiple molecular species coexisting in the same sample. This is because of the fact that, unlike X‐ray crystallography, cryo‐EM does not require crystals, which by definition trap the molecule in a defined state. Other than limitations imposed by the freezing process itself, molecules in a cryo‐EM sample are in a relatively native environment, with the freezing capturing whatever mixture existed in solution. Powerful computational approaches have been developed to sort these species out, effectively “purifying” them in silico (the 3D equivalent of the 2D classification shown in Fig. 2E,F). As a result, cryo‐EM cannot only reveal whether a molecule exists in different oligomeric states (monomers, dimers, etc.) but also whether parts of the molecule can adopt different conformations, an insight into the dynamics of the molecules that can have profound biological implications.

## Cryo‐ET

Cryo‐EM, as described above, relies on having many different views of the same molecule in the sample; without them, a 3D reconstruction cannot be obtained. If one wishes to reconstruct a “unique” object (eg, a subcellular structure of which every instance is different), then all the views needed for the reconstruction must be collected from the same sample. This is precisely what cryo‐ET does: the sample is rotated inside the microscope and images are acquired every few degrees (Fig. 2L). In many ways, cryo‐ET is similar to computerized axial tomography (CAT), although in the latter the subject is kept still while the imaging system is rotated. Like cryo‐EM, cryo‐ET is carried out at liquid nitrogen temperatures.

A major challenge for cryo‐ET is the thickness of the sample; the longer the distance electrons have to travel through the sample, the more likely they are to interact with it more than once, at which point they no longer contribute to the final image in a useful way. A recent development in cryo‐ET addresses this challenge and is ushering in a new era of structural cell biology. In this advance, known as focused ion beam (FIB) milling, a beam of ions is used inside a specialized microscope to slice away layers of the cells down to sections of the right thickness (Fig. 2K). These cells are grown on a grid (Fig. 2J) and frozen just like molecules are for cryo‐EM, (Fig. 2K). In one of its most powerful applications, called correlative light and electron microscopy (CLEM), researchers can express a fluorescently tagged protein of interest inside cells and, then, use a special light microscope operated at cold temperatures to guide FIB milling, leaving just the thin section containing the fluorescent protein in its natural cellular environment (Fig. 2L).

Once a tomogram has been reconstructed (Fig. 2M), it can be mined for data in different ways. The initial one (not shown) involves “segmentation”: identifying boundaries in the (noisy) data to define different cellular entities. If any of the objects in the tomogram appear in multiple copies with similar structure, these smaller regions of the tomogram can be extracted (at which point they are called “sub‐tomograms”) and averaged to improve their signal‐to‐noise ratio, much as it is done in single‐particle cryo‐EM, although in cryo‐ET this sorting is done directly in 3D (Fig. 2N,O). If the resolution of the final structures (“sub‐tomogram averages”) is good enough, molecular models can be built into them, either using the same approaches used in cryo‐EM, or others specialized for cryo‐ET, where resolutions are typically significantly lower. A recent in‐depth description of cryo‐ET, its potential and challenges, can be found in Turk and Baumeister.^33^

### What Can Be Learned at Different Resolutions?

We wanted to provide those less familiar with structural biology with a quick guide to what can be learned from an experimental cryo‐EM/cryo‐ET map, depending on its resolution. At resolutions in the 10‐15 Å range, the shapes of domains are well resolved; if molecular models (experimental or homology‐based) are available, one should be able to dock them unambiguously into the density. At resolutions in the 4‐10 Å range, secondary structure elements become gradually apparent; α‐helices are resolved first (8‐10 Å), followed by β‐sheets, with individual β‐strands being resolved last (~4.5 Å). Bulky amino acid side chains become visible at resolutions around 4 Å, with many side chains showing up (at least partially) once a map reaches a resolution of 3.5 Å. Beyond 3 Å, side chains can be easily recognized, making the building of accurate molecular models much simpler.

## The Structure of Cytosolic LRRK2

Using cryo‐EM, Deniston and colleagues obtained a 3.5 Å‐resolution structure of the carboxy‐terminal half of LRRK2, containing the ROC, COR, kinase, and WD40 domains (that we will refer to here as LRRK2^RCKW^) (Fig. 1B; Video 1).^29^ LRRK2^RCKW^ has an overall J‐shape; the ROC domain constitutes the short end, the COR domain (composed of COR‐A and COR‐B subdomains) makes the turn, and the kinase and WD40 domain line up along the long end. A consequence of this arrangement is that the ROC GTPase and the kinase come in close proximity despite their being separated along the linear sequence of the protein (Fig. 1B). This proximity could account for the known biochemical crosstalk between the kinase and the GTPase.^34^, ^35^, ^36^, ^37^, ^38^, ^39^, ^40^, ^41^, ^42^

Two other features of the structure are worth mentioning here, one because it will become important in the following sections, and another for being unusual. The first feature relates to the kinase domain itself. Kinases are bi‐lobed structures with their active sites—where phosphoryl transfer from adenosine triphosphate (ATP) to a substrate takes place—located in a crevice between the two lobes, a smaller amino‐terminal lobe (N‐lobe) and a larger carboxy‐terminal one (C‐lobe). When ATP is bound, the two lobes come closer together into the active conformation. The kinase of LRRK2^RCKW^, whose structure was solved in the absence of any ligands for the kinase, is in an open or inactive conformation. The second and unusual feature of LRRK2^RCKW^ is found at the very end of the protein and consists of a long α helix that follows the WD40 domain (Fig. 1B; Video 1). This helix runs along the backside of the kinase (ie, opposite to where the active site is located), making close interactions with both lobes. The very end of this helix is found at a place where the kinase and COR domains interact with each other. Interestingly, at least one amino acid near the end of this carboxy‐terminal helix is a known phosphorylation site.^43^ It is tempting to speculate that this carboxy‐terminal helix will be an important element, potentially as a hub involved in controlling the conformation of the kinase. This would be consistent with the early observation that truncating LRRK2's carboxy terminus inactivates the kinase.^44^

Deniston and colleagues^29^ also obtained a number of cryo‐EM structures of LRRK2^RCKW^ dimers, albeit at lower resolutions. These structures showed that LRRK2^RCKW^ could form either COR:COR or WD40:WD40 dimers (Fig. 1C), at least at the high protein concentrations used to prepare the grids for cryo‐EM. Although COR‐mediated dimerization had already been proposed for LRRK2 based on the crystal structure of a bacterial Roco protein containing ROC and COR domains,^23^, ^24^ the dimer of the bacterial Roco protein involves interactions between both the COR and ROC (GTPase) domains, which led to the proposal that the effect of the nucleotide state of the ROC domain on dimerization is mediated by this direct ROC‐ROC interaction. However, the ROC domains are far apart in the COR:COR LRRK2^RCKW^ dimer, suggesting that regulation of LRRK2's dimerization by the GTPase^35^, ^38^ must involve a more indirect mechanism.

## The Structure of Microtubule‐Associated LRRK2

**FIG 3:**
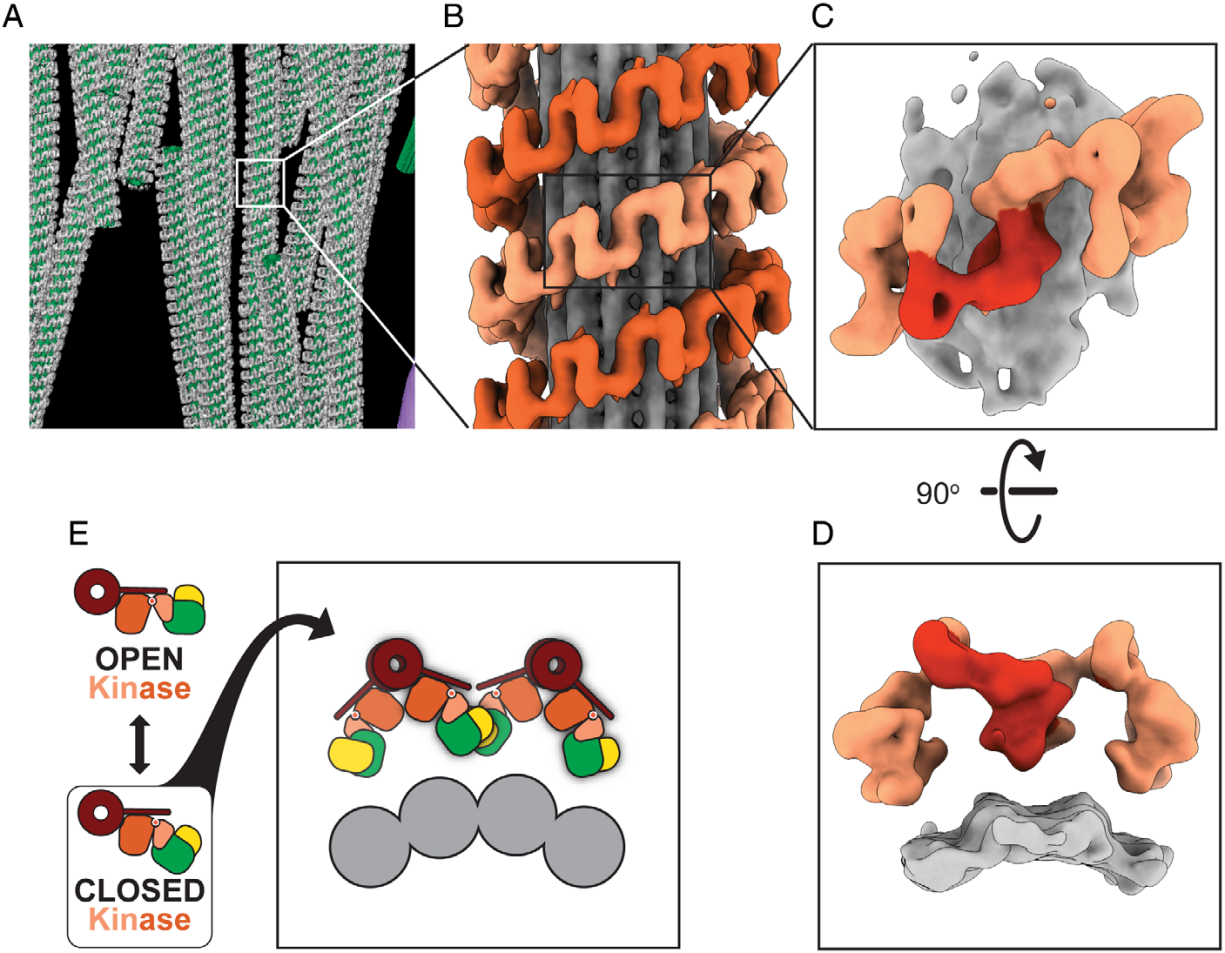
Cryo‐ET of microtubule‐associated LRRK2. (**A**) Tomogram of microtubule (green)‐associated LRRK2 (gray) in cells. (**B**) Sub‐tomogram average of microtubule (gray)‐associated LRRK2 (shades of orange). (**C**) Further sub‐tomogram averaging led to a 14 Å structure of microtubule‐associated LRRK2. (**D**) A 90° rotation of the structure in (**C**) shows a lack of density between the LRRK2 filaments (shades of orange) and the microtubule (gray). (**E**) Docking the structure in Fig. 1B into the structure in Fig. 3C suggested that LRRK2's kinase domain (orange) needed to be in a closed conformation to form filaments around microtubules (gray).

In a tour‐de‐force of cryo‐ET, Watanabe and colleagues^20^ obtained a 14 Å‐resolution structure of microtubule‐associated LRRK2 in cells (Fig. 3A–D). At this resolution, protein domains can be resolved, and the map can be combined with other structural, biophysical, and biochemical data, in what is known as integrative modeling,^45^ to generate a set of molecular models that can explain the density.

In this work, the authors grew cells overexpressing fluorescently labeled LRRK2 carrying the PD‐linked mutation I2020T, which is known to increase LRRK2's association with microtubules.^15^ Because of its fluorescence, LRRK2 associated with microtubules could be located using light microscopy after the cells had been frozen. The researchers used FIB milling to generate thin sections containing microtubules and LRRK2, and imaged them in the electron microscope. Using this approach, Watanabe and colleagues^20^ generated several 3D reconstructions of microtubules with LRRK2 forming filaments around them (Fig. 3A,B). Because of the repetitive nature of these filaments, they could split each LRRK2‐decorated microtubule into many copies of a minimal repeating unit containing a section of microtubule and about four LRRK2 monomers (sub‐tomograms, as in Fig. 2N). By aligning and averaging together all of those copies (as in Fig. 2O), they obtained a final structure of LRRK2 bound to microtubules at 14 Å resolution (Fig. 3C,D).

The structure obtained by Watanabe and colleagues^20^ showed that LRRK2 wraps around microtubules as a right‐handed double‐stranded helix (Fig. 3B). This was an unusual and unexpected arrangement, because microtubules are left‐handed helices, therefore resulting in a symmetry mismatch between them and the LRRK2 helices. The mismatch has functional implications at the molecular level as it suggests that different LRRK2 monomers along a filament could “see” different features on the microtubule surface.

Even though the cryo‐ET structure shows well‐defined density for both LRRK2 and the microtubule, there is no clear density connecting the two (Fig. 3D). Given that the structure was obtained by averaging multiple copies, this absence of density suggests that the connections between LRRK2 and the microtubule vary, and therefore, were averaged out during sub‐tomogram averaging. This further supports the idea that the symmetry mismatch between LRRK2 and the microtubule leads to more than one type of interaction between the two.

Finally, the density corresponding to LRRK2 in the structure of the microtubule‐bound filaments could be accounted for by modeling only the carboxy‐terminal half of LRRK2, the same four domains that were present in the cryo‐EM structure of LRRK2^RCKW^, although the LRRK2 present in the cells was the full‐length protein. This is likely a result of a flexible amino‐terminal half of LRRK2 being averaged out during the reconstruction process. In fact, a small bit of density could be seen adjacent to the ROC domain, right where the LRR domain is expected to connect to it.^20^ The modeling was confirmed when the high‐resolution structure of LRRK2^RCKW^ was docked into the cryo‐ET map (Fig. 3E).^29^ An important and related observation was that microtubule‐associated filaments could be reconstituted in vitro using only purified LRRK2^RCKW^ and microtubules.^29^ These observations suggest that the carboxy‐terminal half of LRRK2 is sufficient for binding to and oligomerizing on microtubules.

Docking the high‐resolution structure of LRRK2^RCKW^ into the cryo‐ET map to build a molecular model of the filaments led to an important insight into how their formation is regulated. As we mentioned earlier, the kinase portion of LRRK2 was in an open conformation in the high‐resolution structure of LRRK2^RCKW^. This structure could not be fitted directly into the cryo‐ET reconstruction of the filaments without some clashes at one of the interfaces between LRRK2^RCKW^ monomers.^29^ However, the majority of these clashes could be resolved by modeling the kinase in a closed conformation.^29^ Because the kinase is located in a central position in LRRK2^RCKW^ (Fig. 1B), where it can act as a “hinge,” its closing makes the entire LRRK2^RCKW^ more compact (Fig. 3E). The markedly improved fitting led the authors to propose that LRRK2's kinase had to be in its closed conformation for LRRK2 oligomers to form on microtubules (Fig. 3E). In the next section, we discuss how this hypothesis was tested and its implications for the role of LRRK2 in cells.

## What Is the Function of Microtubule‐Associated LRRK2 in Cells?

**FIG 4:**
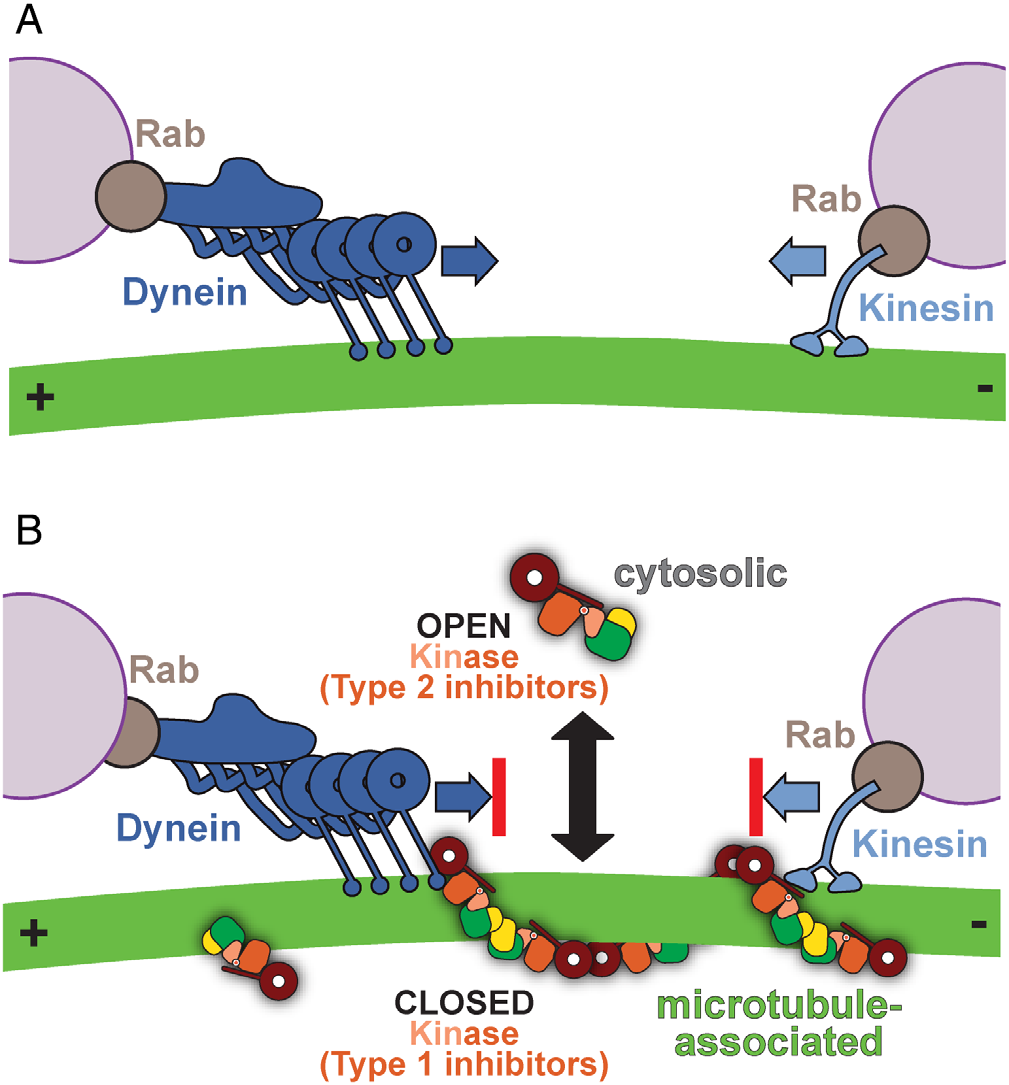
LRRK2^RCKW^ acts as a roadblock for dynein and kinesin motors. (**A**) Dynein and kinesin are microtubule‐based motors that transport many cargos, including membranes marked by Rab GTPases. (**B**) Type 1 kinase inhibitors promote the closed conformation of LRRK2's kinase, favoring microtubule‐association, whereas type 2 kinase inhibitors promote the open conformation of LRRK2's kinase. Dynein and kinesin motility is blocked by LRRK2 in the presence of type 1 kinase inhibitors, whereas type 2 kinase inhibitors rescue these LRRK2^RCKW^ roadblocks.

Many cellular components, from larger protein and ribonucleoprotein complexes to organelles, are too large to rely on diffusion to reach their target location in cells on biologically relevant time scales. Evolution has solved this problem with the molecular motors dynein and kinesin, which transport cargo along microtubules for long distances^46^ (Fig. 4A). Microtubules are polar filaments, meaning they have an intrinsic directionality. They are also dynamic, constantly growing and shrinking. Their slower growing ends (the “minus” ends) are generally located near a microtubule organizing center, often close to the nucleus, whereas the faster growing ones (the “plus” ends) are typically located near the cell periphery. Dynein and kinesin can read the polarity of microtubules to move unidirectionally, with dynein transporting cargo toward microtubule minus ends, and most kinesins in the opposite direction. Both dynein and kinesin walk in a fairly anthropomorphic manner, generally putting “one foot in front of the other.” Kinesin walks in a very straight line,^47^ and is, therefore, easily blocked by obstacles, whereas dynein, larger and more flexible, can meander and navigate obstacles better.^48^, ^49^, ^50^

Deniston and colleagues^29^ tested whether LRRK2^RCKW^ could block the motion of dynein and kinesin along microtubules using “single‐molecule” in vitro assays. In these experiments, microtubules are chemically attached to a coverslip, which is attached to a slide, forming a very small liquid‐containing chamber in between. Purified, fluorescently labeled motors (either dynein or kinesin) are then flowed in, and they are observed using total internal reflection fluorescence microscopy. With this technique, researchers can visualize individual motors as they walk along microtubules and measure their motile properties. Using this approach, the authors discovered that even low concentrations (low nanomolar) of LRRK2^RCKW^ significantly blocked the motors; this was even true for dynein,^29^ despite its known ability to circumvent obstacles.^51^

This observation had both biological and practical implications. Biologically, the fact that small amounts of LRRK2 can prevent molecular motors from moving along microtubules (and therefore, presumably, from carrying their cargo) raises intriguing questions as to what function a pool of microtubule‐associated LRRK2 might serve. In practical terms, it provided a tool to test the hypothesis that the closed conformation of LRRK2's kinase is necessary for it to oligomerize on microtubules. Specifically, the motors could be used as sensitive probes to detect, and measure, binding of LRRK2 to microtubules. The hypothesis predicted that forcing the kinase into an open conformation, which should prevent binding to microtubules, should rescue dynein and kinesin from being blocked by LRRK2 (Fig. 3E).

One way to control the conformation of a kinase is by using small‐molecule kinase inhibitors. Kinases are some of the most “druggable” proteins in the human proteome, with 62 Food and Drug Administration (FDA)‐approved compounds currently in use in the clinic to treat cancer, autoimmune, and inflammatory disorders.^52^ Although most inhibitors prevent the kinase from binding ATP, the details of how they bind to the kinase's active site determine whether the kinase adopts an open or closed conformation; generally, “type 1” inhibitors result in a closed conformation, whereas “type 2” inhibitors lead to an open one.^53^ This is the approach that Deniston and colleagues^29^ took. In support of their hypothesis, LRRK2^RCKW^ could no longer block dynein or kinesin when a type 2 inhibitor was added to their single‐molecule motility assay. Conversely, type 1 inhibitors, which should favor the closed conformation and, therefore, formation of filaments, did not prevent the motors from being blocked by LRRK2^RCKW^ (Fig. 4B). Similar results were obtained in cells overexpressing a fluorescently labeled version of LRRK2; treating cells with a type 2 inhibitor decreased LRRK2's ability to form microtubule‐associated filaments, whereas, as had been shown previously,^34^, ^54^ type 1 inhibitors enhanced filament formation.^29^ Several LRRK2‐specific type 1 inhibitors have been developed, including MLi‐2^55^, ^56^ and LRRK2‐IN‐1,^57^ the type 1 inhibitors used in the study by Deniston and colleagues.^29^ Other LRRK2‐specific type 1 inhibitors include DNL151, pursued by Denali Therapeutics and Biogen; this inhibitor completed phase 1b clinical trials late last year. Although the structure of DNL151 has not been publicly disclosed, based on an examination of the patent literature it is potentially a pyrimidinyl‐4‐aminopyrazole compound similar to the Genentech type 1 inhibitors GNE‐0877 and GNE‐9605.^58^ The type 2 inhibitors used by Deniston and colleagues^29^ —ponatinib^59^ and GZD824^60^—are not LRRK2‐specific and were developed to target BCR‐ABL. Although there are currently no LRRK2‐specific type 2 inhibitors available, the work by Deniston and colleagues^29^ highlights the importance of pursuing them because it is conceivable that even a very low level of blockage of microtubule‐based transport could be detrimental given the chronic nature of small molecule treatment of PD.

## The Road Ahead

The work reviewed here, although providing important structural and functional insights into LRRK2 and its interaction with microtubules, is just the tip of the iceberg when it comes to understanding LRRK2's complexity at the molecular and cellular levels, as well as its relationship to PD. Here, we will briefly discuss what we see as some of the most immediate challenges, focusing on questions that require structural insights.

### How Does LRRK2 Recognize its Substrates?

As mentioned earlier, LRRK2 phosphorylates a subset of Rab GTPases that mark cargos that are transported along microtubules.^9^, ^17^ Given that all of the most common PD‐linked mutations in LRRK2 lead to activation of its kinase, one or more Rabs may be the molecular link between LRRK2's enzymatic activity and the cellular basis of the disease. Structures of LRRK2‐Rab complexes will reveal how the Rabs are recognized, potentially enabling the ability to control LRRK2 binding to specific Rabs in gene‐edited cells and ultimately determine which Rabs are linked to PD.

### How Is LRRK2's Activity Regulated?

The available data already point to the complex regulation of LRRK2. Its kinase activity has been shown to be modulated by a number of different factors: the monomeric or dimeric state of the protein^14^, ^19^, ^61^, ^62^, ^63^, ^64^; the nucleotide state of the ROC GTPase^34^, ^35^, ^36^, ^37^, ^38^, ^39^, ^40^, ^41^, ^42^; phosphorylation of residues throughout the protein, both near and far away from the kinase^10^, ^42^, ^43^, ^65^, ^66^; and cellular localization.^14^, ^34^, ^65^, ^67^, ^68^ Structural information will be essential to understand how all of these factors affect LRRK2's enzymatic and biological activities, and how these are disrupted by PD‐linked mutations.

### What Controls LRRK2's Sub‐Cellular Localization?

What is the function of LRRK2 in its different reported sub‐cellular localizations: cytosolic, membrane‐ and microtubule‐associated? What regulates its partitioning? An important component of understanding how hyperactivation of LRRK2 leads to PD will be determining where in the cell and in what cell types that increase in activity has its effect. To do that, particular sub‐cellular pools of LRRK2 will need to be disrupted, which in turn requires understanding, mechanistically, what controls its distribution and how LRRK2 interacts with specific components of those sub‐cellular locales (eg, membranes or microtubules). In addition to characterizing the specific interactions between LRRK2 filaments and microtubules, determining how LRRK2 is recruited to and interacts with membranes is an important future direction.

### How Do PD Mutations Affect LRRK2's Activity and Cellular Function?

Of the most common PD‐linked LRRK2 mutations, only two—G2019S and I2020T—are found in the active site of the kinase. A few others are located at the interface between the ROC and COR domains (Fig. 1B). Although the structure of LRRK2^RCKW^ showed that ROC/COR‐A are close in space to the kinase (specifically its C‐lobe) (Fig. 1A,B),^29^ it did not provide a molecular explanation for how that activation would take place. Given the many factors involved in regulating LRRK2 we have described here, it is likely that only structures of full‐length LRRK2 carrying PD‐linked mutations will reveal how they activate the kinase, and whether they share a common mechanism for doing so.

### Targeting LRRK2 for Therapeutics

Ever since LRRK2 was cloned^3^ and its hyperactive kinase activity was linked to disease,^7^, ^8^, ^9^ inhibitors of LRRK2's kinase activity have been sought after as a promising therapeutic route for treating PD. Several LRRK2‐specific type 1 kinase inhibitors have been developed^55^, ^57^, ^58^, ^69^, ^70^ and one completed phase 1b clinical trials (clinicaltrials.gov). The work discussed here suggests that developing LRRK2‐specific type 2 kinase inhibitors may be important, as type 1 inhibitors may have the unwanted effect of promoting binding of LRRK2 to microtubules and generating roadblocks that block the movement of intracellular cargos transported by dynein and kinesin motors. Structures of LRRK2 bound to LRRK2‐specific inhibitors will be important for targeted medicinal chemistry approaches to fine tune these inhibitors for specificity and the ability to cross the blood–brain barrier.

## Structure of Full‐Length LRRK2

Both LRRK2 structures reviewed here were missing the N‐terminal half of the protein, either because it was absent from the molecule altogether (in the cryo‐EM structure of soluble LRRK2^RCKW^) or because it was disordered and averaged out during image processing (in the case of the cryo‐ET structure of microtubule‐associated full‐length LRRK2 in cells). An exciting new paper reporting cryo‐EM structures of full‐length LRRK2 monomers and dimers was published after this perspective had been reviewed.^71^ Although the timing prevented us from discussing that work in detail and including it in our figures, we wanted to highlight the most salient features and implications of those structures here. (1) The amino‐terminal domains—armadillo (ARM), ankyrin (ANK) and leucine‐rich repeats (LRR)—form a long extension, with only the ANK and LRR interacting with the catalytic half of LRRK2. (2) The LRR drapes over the kinase domain, occluding its active site in a way that would prevent substrates (such as Rabs) from accessing it. (3) The position of the amino‐terminal half in the structure would prevent formation of the WD40‐mediated dimerization interface observed in the microtubule‐associated filaments of LRRK2. (4) A structure of a LRRK2 monomer carrying the G2019S mutation was very similar to that of the wild‐type protein; both showed the kinase domain in the open or inactive conformation. (5) A structure of a dimer of full‐length LRRK2 shows the monomers interacting via their COR domains, the same interface identified in the microtubule‐associated filaments^20^ and in one of the soluble forms of the LRRK2^RCKW^ dimer.^29^ As predicted, mutating this interface abolished formation of microtubule‐associated filaments in cells.

## Author Roles

A.E.L. and S.L.R.P. wrote and edited the manuscript.

## Full financial disclosure for the previous 12 months

Andres E. Leschziner and Samara L. Reck‐Peterson received funding from the Michael J. Fox Foundation, the Aligning Science Across Parkinson's, and the National Institutes of Health (R01 GM092895, R01 GM107214, R35 GM130389, and R01 GM132720 to Leschziner, and R01 GM121772, and R35 GM141825 to Reck‐Peterson). Andres E Leschziner received a grant from the National Science Foundation (1759826). Samara L. Reck‐Peterson received funding from the Howard Hughes Medical Institute.

## Supporting information


**Video 1** 3.5 Å Cryo‐EM structure of LRRK2^RCKW^. This video shows an overview of the 3.5 Å cryo‐EM structure of cytosolic LRRK2^RCKW^. The structure was colored according to the conventions introduced in Fig. 1B. The only amino acid side chains shown in this video, in black, are those corresponding to the sites of the most common PD‐linked mutations shown in Fig. 1B. I2020, indicated with square brackets, is not present in our model because the corresponding part of the cryo‐EM map lacks well‐defined density (a sign of flexibility in the underlying structure). The GDP nucleotide bound to the ROC domain is shown in blue.

## Data Availability

Data sharing not applicable ‐ no new data generated.
